# Assessing emerging contaminants in Antarctic benthic marine fauna: a dual mass spectrometry investigation combining targeted and suspect screening approaches

**DOI:** 10.1007/s00216-026-06512-3

**Published:** 2026-04-28

**Authors:** Julia Gambetta Vianna, Erica Ceccardi, Barbara Benedetti, Jung-Keun Oh, Marina Di Carro, Sarah Pizzini, Emanuele Magi

**Affiliations:** 1 Department of Chemistry and Industrial Chemistry, University of Genoa, Via Dodecaneso 31, 1646 Genoa, Italy; 2 Department of Environmental Sciences, Informatics and Statistics, Ca’ Foscari University of Venice, Via Torino 155, Venice, Mestre Italy; 3 Environmental Health Research Division, National Institute of Environmental Research, Hwangyeong-Ro 42, Seo-Gu, Incheon, 22689 Republic of Korea; 4 Institute for Marine Biological Resources and Biotechnology, National Research Council (CNR-IRBIM), Largo Fiera Della Pesca 2, 60125 Ancona, Italy

**Keywords:** Environmental pollution, Microcontaminants, Polar environment, Marine bioindicators, Liquid chromatography-mass spectrometry, Non-target mass spectrometry

## Abstract

Antarctica hosts a highly endemic and diverse benthic marine fauna. Despite this biodiversity, the Antarctic marine food web remains structurally simple, rendering the ecosystem particularly vulnerable to environmental stressors. Benthic organisms, due to their sedentary nature, long lifespans, and close interaction with the sediment-water interface, are widely regarded as effective sentinels of ecological change. In this study, we extended a previously validated QuEChERS-based extraction protocol, originally developed for *Adamussium colbecki* organisms, to assess its applicability across additional Antarctic benthic taxa, including *Sphaerotylus antarcticus*, *Odontaster validus*, *Trematomus bernacchii*, and *Laternula elliptica*. The extraction method was used in combination with LC-MS/MS analysis for the determination of emerging contaminants in both targeted and suspect screening modes. Method performance was evaluated for 23 targeted emerging contaminants (ECs), yielding recovery rates of 58–116% and matrix effects between 62 and 108% for most compounds, confirming the method’s suitability for taxonomically diverse matrices. Samples collected during Antarctic expeditions from 2018 to 2022 revealed the presence of multiple ECs, including perfluorooctanoic acid (PFOA), caffeine, pharmaceuticals and personal care products (PPCPs), and UV filters. Complementarily, a preliminary suspect screening via high-resolution mass spectrometry was attempted, revealing the potential presence of a broader spectrum of drugs, PPCPs, and lifestyle-related compounds in all studied species. This work represents one of the first applications of a QuEChERS-based analytical framework for ECs detection in Antarctic marine fauna, offering a reliable approach for long-term contaminant monitoring in one of the planet’s most fragile ecosystems.

**Supplementary Information:**

The online version contains supplementary material available at https://doi.org/10.1007/s00216-026-06512-3.

## Introduction

Antarctica, owing to its long-standing geographical isolation and absence of native human populations, has remained one of the least anthropogenically influenced regions on Earth. The continent’s present position at the South Pole was established approximately 45 million years ago and remained biogeographically isolated by the Antarctic Circumpolar Current [1]. Within this physically and climatically extreme environment, a rich and endemic marine fauna has evolved, particularly within benthic communities, with over 4100 species of macroinvertebrates described [2]. Despite this taxonomic richness, the Antarctic marine food web remains structurally simple, primarily due to intense environmental constraints such as prolonged periods of darkness, ice coverage, and low primary productivity. These factors limit trophic complexity and redundancy, rendering the system ecologically fragile and highly susceptible to perturbation [2, 3].

Benthic species, defined as organisms inhabiting the seafloor or its immediate vicinity, constitute a fundamental component of Antarctic marine ecosystems. Due to their relatively long lifespans, sessile or low-mobility habits, and direct exposure to sediment-water interactions, benthic organisms are widely recognized as sensitive indicators of environmental change [3–5]. Among the benthic biota, sponges, such as the *Sphaerotylus antarcticus*, represent a dominant group in terms of biomass and biodiversity, with over 350 species recorded [6–8]. Other notable benthic species include the sea star *Odontaster validus*, an omnivore exhibiting diverse feeding strategies, from predation and scavenging to herbivory and suspension feeding [9–11]. Similarly, the bivalve *Laternula elliptic*a is a widely distributed and ecologically important infaunal species, often considered a sentinel organism in benthic research due to its sensitivity and ubiquity in Antarctic habitats [12–14]. The emerald notothen *Trematomus bernacchii*, a benthic fish endemic to the Southern Ocean, occupies a pivotal trophic position, preying primarily on benthic invertebrates and mediating energy flow between lower and higher trophic levels [15–17]. Collectively, these organisms form a tightly interlinked ecological network whose structure and function are closely governed by environmental variability and resource inputs.

In recent decades, the intensification of human presence along the Antarctic coastline has introduced new classes of pollutants into these ecosystems [1, 18]. Long-range transport (LTR) and local anthropogenic activities are possible pathways for the entry of emerging contaminants (ECs) into the marine environment [19, 20]. These compounds, encompassing endocrine-disrupting chemicals, pharmaceuticals, and personal care products (PPCPs), are of particular concern in polar regions due to their potential persistence under cold conditions, their potential for bioaccumulation, and their capacity for trophic transfer through simplified food webs [19, 21]. The detection of ECs in Antarctic seawater, sediments, and biota is essential to monitor the environmental fate, toxicity, and long-term ecological implications [22, 23].

However, the study of ECs in polar biota presents substantial analytical challenges due to the complexity of biological matrices, often characterized by high lipid content and interfering substances [24, 25]. Conventional extraction techniques, although effective, are time-consuming, resource-intensive, and often characterized by low selectivity, leading to the co-extraction of interferent species and consequent non-negligible matrix effects. The QuEChERS (Quick, Easy, Cheap, Effective, Rugged, and Safe) method, originally developed for pesticide analysis in food matrices, has emerged as a promising alternative for multi-residue contaminant analysis in environmental biota. Nevertheless, its direct application to polar species requires rigorous optimization to account for matrix-specific interferences, especially when a broad range of target analytes are considered [24, 26–29]. In this context, the use of sensitive, selective, and accurate instrumental techniques such as liquid chromatography coupled to mass spectrometry (LC-MS) plays a pivotal role.

In this study, an expansion of a previously optimized QuEChERS extraction method developed for analysis of ECs in *Adamussium colbecki*, combined with a liquid chromatography coupled with tandem mass spectrometry (HPLC-MS/MS) analysis, was undertaken, to evaluate its applicability to other representative Antarctic benthic species [25]. Specifically, the method was tested on *S. antarcticus*, *O. validus*, *T. bernacchii*, and *L. elliptica*, covering the key benthic taxa such as sponges, echinoderms, fish, and clams. Method performance was evaluated using standard validation parameters, including recovery rates and matrix effects, as well as inter- and intra-day repeatability. The protocol was then applied to determine the presence of 23 target ECs in samples collected during Antarctic campaigns between 2018 and 2022. Additionally, a suspect screening analysis (SSA) using high-resolution mass spectrometry (HRMS) was performed as a preliminary assessment on the occurrence of over 2000 suspect compounds, including PPCPs and drugs. This study represents one of the first reported applications of a QuEChERS-based approach for the analysis of ECs in Antarctic marine biota, establishing a framework for long-term environmental monitoring and assessment of contaminant impacts in one of Earth’s most vulnerable ecosystems.

## Materials and methods

### Standards and reagents

Analytical standards were sourced from multiple commercial suppliers and were of high purity grade ≥ 97%. Caffeine (CAFF), ketoprofen (KETO), naproxen (NAP), theobromine (THB), and diclofenac (DCF) were procured from Fluka Analytical (Saint Gallen, Switzerland), while salbutamol (SLBT) was obtained from Alfa Aesar (Haverhill, MA, USA). The remaining compounds were purchased from Sigma-Aldrich (St. Louis, MO, USA), including paraxanthine (PRX), theophylline (TFL), carbamazepine (CBZ), hydrochlorothiazide (HCTZ), benzophenone-3 (BP-3), octyl dimethyl p-aminobenzoate (OD-PABA), ethylhexyl methoxycinnamate (EHMC), octocrylene (OC), perfluorooctanoic acid (PFOA), perfluorooctane sulfonate (PFOS), acesulfame K (ACS), sucralose (SCL), bisphenol A (BPA), estrone (E1), β-estradiol (E2), 17α-ethinylestradiol (EE2), ibuprofen (IBU), gemfibrozil (GEM), furosemide (FRSM), metoprolol (MTPL), clenbuterol (CLBT), terbutaline (TRBT), atenolol (ATN), ethylhexyl salicylate (EHS), nicotine (NCT), cocaine (COCA), omethoate (OMT), daminozide (DMNZ), 2,4-dichlorophenoxyacetic acid (2,4-D), chloramphenicol (CMPH), metformin (MTF), mepiquat (MPQ), chlormequat (CMQ), and triclosan (TCS). Stock solutions of all analytes were prepared in either pure methanol or methanol-water (1:1) mixture, depending on solubility.

Reagents for extraction and clean-up were also obtained from various suppliers. Magnesium sulfate was purchased from Carlo Erba Reagents (Rodano, MI, Italy), and sodium chloride from Sigma-Aldrich. Dispersive solid-phase extraction (d-SPE) sorbents included end-capped C18 silica, acquired from Supelco (Bellafonte, PA, USA) and primary secondary amine (PSA) sorbents from Phenomenex (Torrance, CA, USA). MS-grade solvent acetonitrile (ACN) and acetic acid (AA) used as a pH regulator were supplied by Merck (Darmstadt, Germany). Ultrapure water was produced using a Millipore Q-Gard purification system equipped with a 0.22-µm Millipak filter (Millipore, Watford, UK).

### Sample collection and storage

Specimens from five benthic species (*A. colbecki*, *O. validus*, *T. bernacchii*, *S. antarcticus*, and *L. elliptica*) were collected by Korean researchers near Jang Bogo Station (South Korea) during ethically conducted Antarctic scientific campaigns. Sampling took place in Terra Nova Bay, a glacially influenced inlet located in the southwestern Ross Sea, bounded by Cape Washington to the north and the Drygalski Ice Tongue to the south. The bay, approximately 80 km long and 30 km wide, features a continental shelf with an average of 450 m and maximum depths reaching 1100 m in the Drygalski Basin [30].

Precise sampling sites, species collected, and collection years are detailed in Table 1. Collection of all species except *L. elliptica* occurred at depths ranging from 20 to 30 m, where the benthic substrate was characterized by granitic bedrock interspersed with patches of coarse sand and gravel. *L. elliptica* was collected near Gondwana Station.

**Table 1:** Specimen, sampling sites, and collection years of the analyzed samples

Specimen	Sampling year	Sampling coordinates
*O. validus*	2018	74°37′ S 164°13′ E
*T. bernacchii*	2021	74°37′ S 164°14′ E
*S. antarcticus*	2019	74°37′ S 164°14′ E
*L. elliptica*	2022	74°38′ S 164° 13′ E
*A. colbecki*	2018	74◦38′ S 164◦13′ E
*A. colbecki*	2019	74◦38′ S 164◦13′ E

The samples, obtained during the 2018 and 2022 expeditions, were immediately sealed in polyethylene bags upon collection and stored at −80 °C to maintain integrity until analysis.

Prior to laboratory processing, specimens were thawed at 4 °C. Morphometric data, including total length and wet weight, were recorded. Soft tissues were carefully dissected and thoroughly rinsed with ultrapure water to remove adhering sediments and debris. The cleaned tissues were immediately stored under cryogenic conditions in vapor-phase liquid nitrogen (boiling point, − 196 °C) prior to pretreatment. Samples were subsequently cryogenically homogenized by three successive grinding cycles using a planetary ball mill (Fritsch Planetary Mill, Fritsch, Idar-Oberstein, Germany), followed by freeze-drying to ensure sample integrity, preservation, and analytical stability [31].

### Extraction and clean-up

The QuEChERS-based extraction protocol applied in this study was optimized in a previous work by Gambetta Vianna et al. (2025). Briefly, biota tissue (100 mg) was extracted with 12 mL of a 50:50 (v/v) ACN:H_2_O mixture, followed by salting-out-assisted phase separation, using 3.4 g of MgSO_4_ and 0.9 g of NaCl, and centrifugation. Two milliliters of the resulting ACN layer was subjected to dispersive solid-phase extraction (d-SPE) clean-up using 85 mg of PSA and 100 mg of C18 sorbents. After vortexing for 1 min and a second centrifugation, 1 mL of the supernatant was evaporated under a gentle nitrogen stream and reconstituted in the same volume of 50:50 (v/v) MeOH:H_2_O [25].

Prior to instrumental analysis, the extract was passed through a 0.2-µm PTFE syringe filter and 5-fold diluted with the same MeOH:H_2_O solution. The final extract was subjected to LC-MS/MS analysis by two different instruments, as described below.

A procedural blank was prepared by applying the same analytical procedure in the absence of the sample. This blank was used to verify the presence of potential contamination in targeted analysis and to eliminate identifications not associated with the sample in non-targeted analysis.

### Targeted HPLC-MS/MS analysis

All analyses were conducted using an Agilent 1200 series liquid chromatography system coupled to a 6430 triple quadrupole mass spectrometer (Agilent Technologies, Santa Clara, CA, USA), equipped with an electrospray ionization (ESI) source. The mass spectrometer operated under the following conditions: nitrogen (N_2_, purity > 98%) was used as the drying gas at a temperature of 300 °C and a flow rate of 11 L min^−1^; the nebulizer pressure was set to 15 psi; and the capillary voltage was maintained at 4000 V. Chromatographic separation was performed using a Supelco Ascentis Express biphenyl column (100 × 2.1 mm, 2.7 µm). The column was maintained at 50 °C and the injection volume was set at 10 µL. The method involved a gradient elution with H_2_O and ACN 0.1% AA, as phases A and B, respectively. The method’s gradient is reported in Table S1.

ESI polarity switching mode was exploited to determine analytes ionizing in both positive and negative modalities with a single chromatographic run.

MS detection was carried out in dynamic multiple reaction monitoring (d-MRM) mode to enhance both sensitivity and selectivity. Quantification was based on the most intense transition (quantifier), while additional transitions (qualifiers) were monitored for compound confirmation. When only a single MRM transition was available for a given analyte, due to insufficient signal intensity, compound identification was achieved by solely comparing retention times with those of analytical standards, with full awareness of the limitations this may entail in terms of confirmation. Specific MS parameters and MRM transitions are detailed in the Supporting Information (Table S3). Data acquisition, qualitative analysis, and targeted quantitative analysis were performed using the MassHunter 10.0 software from Agilent.

### HPLC-HRMS/MS analysis

Suspect screening analyses were performed using ultra-high-performance liquid chromatography coupled to quadrupole time-of-flight mass spectrometry (UHPLC-ESI-QToF). An Agilent 1260 Infinity II UHPLC system (Agilent Technologies, Santa Clara, CA, USA) was coupled to a QToF model 6546 Infinity mass spectrometer, with a nominal resolution of 60,000.

Chromatographic separation was performed using the same Biphenyl column of the targeted method. A different chromatographic method was developed to optimize the simultaneous detection of both targeted and non-targeted contaminants. The column was maintained at 50 °C and the injection volume was set at 10 µL. The method involved a gradient elution with H_2_O and ACN 0.1% AA, as phase A and phase B, respectively. The method’s gradient is reported in Tables S1 and S2. Two separate chromatographic runs were performed using the same method, first acquiring data in positive ionization mode and subsequently in negative ionization mode, to maximize the potential for identification in suspect screening.

Ionization was accomplished by an ESI jet stream source using nitrogen (N₂, purity > 98%) as drying and sheath gas. Drying gas was set at 200 °C with a flow of 12 L min⁻^1^, while sheath gas was set at 350 °C with a flow of 11 L min⁻^1^. Nebulizer pressure was 30 psi. The capillary voltage was set to 4000 V throughout the run in negative ionization mode; regarding the positive ionization mode, the voltage was maintained at 4000 V until 4.1 min and then decreased to 2600 V, to favor the detection of some classes of more hydrophobic analytes. For the same reason, different fragmentor voltages were applied in time-segmented acquisition: 150 V until 7 min and then 80 V in positive ion mode, whereas 115 V was used in negative ion mode. Data-independent acquisition (DIA) was used to obtain high-resolution MS and MS/MS spectra, to avoid any bias in selecting precursor ions for fragmentation. The MS range was set from 50 to 1000 m/z, with 1.5 spectra/s acquisition rate. For the MS/MS experiments, two voltages (20 and 40 V) in the collision cell were applied in consecutive experiments, to obtain the most informative collision-induced fragmentation. These collision energies were those more frequently observed in the MS/MS spectra from the library used for tentative identifications.

Data acquisition and qualitative analysis were performed using The MassHunter 10.0 software from Agilent.

### HRMS data processing for suspect screening analysis

While the QqQ analysis offers exceptional sensitivity and selectivity, its primary limitation is its inability to perform non-target screening [32].

Preliminary insights on the samples’ unknown contamination were gained through a suspect screening approach. A careful analysis of the HRMS-DIA data was carried out with the aid of the Quant Screener Agilent Tool. To this aim, a spectral library comprising accurate masses of precursor ions, isotopic patterns, and MS/MS spectra at different collision energies was used to select precursor and product ions to be searched within the overall acquired MS (MS1) and MS/MS spectra (MS2), thus attempting the identification of unknowns. The library for positive ionization mode consisted of 1761 compounds, of which 1387 included MS/MS spectra, while the one for negative ionization mode had 1163 compounds, all including MS/MS spectra. The precursor ions considered were the [M+H]^+^, [M-H]^−^, and [M+CH_3_COO]^−^. The data analysis workflow comprised several sequential steps, starting from raw chromatograms. First, blank filtering was applied by removing signals in the samples with intensities lower than or equal to five times the blank signal. Subsequently, identifications associated to peaks with a deviation greater than 30% in areas between replicates were excluded, and retention time correspondence was verified. A mass accuracy threshold of 5 ppm was applied for precursor ion matching. Also, isotopic patterns were compared with database entries by evaluating accurate masses, peak spacing, and expected relative intensities. Then, a chemical formula was assigned only when both accurate mass and isotopic pattern met the defined acceptance criteria. As this study represents a preliminary non-target investigation, no advanced deconvolution of DIA data was performed. Instead, the analysis was based on the screening of precursor ions and associated diagnostic fragments within the overall spectral profiles (MS1 and MS2). In this framework, MS/MS spectra are not related to specific precursors, while fragment assignment relies on the co-occurrence of signals within the same retention time window. In other terms, the overlap of the elution profiles related to extracted ion currents of precursor and fragment ions is verified and expressed as coelution score, which is mathematically calculated by a proprietary Agilent algorithm. Tentative identification, when product ions were detected, was supported by a scoring system implemented in the Agilent MassHunter Quant Screener software, which integrates multiple criteria: (i) the detection of a minimum number of fragment ions (observed in MS2) consistent with a given precursor, (ii) the mass accuracy of both precursor and fragment ions, and (iii) the coelution of precursor and fragment signals (considered acceptable if the coelution score is > 70%). Furthermore, signal-to-noise ratio thresholds of 10 for precursor ions and 3 for product ions were applied. In cases where no MS/MS information was available or no matching product ions were found in the libraries, identification was based solely on accurate mass and isotopic pattern, potentially resulting in multiple candidate annotations due to the presence of isomeric species. Schymanski confidence levels were assigned based on the available information.

A PCA was performed using Chemiometrics Agile Tool (CAT) after autoscaling areas related to level 2 identified chemicals [33].

### Method performance

The analytical performance of the method was evaluated by assessing key validation parameters, including linearity within the concentration working range, accuracy, precision, and the limits of detection (LOD) and quantification (LOQ).

Accuracy (evaluated as recovery and matrix effects) was assessed through recovery experiments using biota tissue samples spiked with known concentrations of the target analytes, performed on two non-consecutive days (total *n* = 6 replicates). Precision was evaluated by calculating the relative standard deviation (RSD%) of replicate applications of the overall method. Intra-day precision was based on three replicates analyzed within a single day, whereas inter-day precision was determined using six replicates across different days. Given the logistical difficulties and limited quantity of Antarctic biota available, the number of replicates was necessarily constrained.

Upon verification of matrix effects, method linearity was assessed by constructing external calibration curves over a concentration range from 0.1 to 50 ng mL^−1^, and by calculating the determination coefficient (*R*^2^).

The LOD was determined experimentally in all matrices by analyzing non-spiked and spiked samples at known concentrations and by looking at the quantifier MRM transition of the target compounds. The entity of the noise signal in each matrix was evaluated by considering regions of the chromatogram at the same retention time and the width of the peak in the spiked matrix. In case the analyte was detected in the non-spiked sample, regions adjacent to the peak were chosen. Then, LOD and LOQ concentrations were determined as the peak areas giving 3 and 10 S/N ratios, respectively, by exploiting a calculation described in a previous work [34]. This rigorous evaluation ensured a conservative and realistic evaluation of the method sensitivity in the matrix.

## Results and discussion

Although the QuEChERS-based method previously optimized for *A. colbecki* demonstrated satisfactory performance for only 18 out of the 40 selected analytes, its broader applicability was systematically evaluated by testing all 40 compounds in the current study. These analytes were selected to encompass a wide chemical spectrum, including pesticides, perfluorinated compounds, UV filters, lifestyle compounds, and PPCPs.

Method applicability to these benthic matrices was assessed by determining R% and ME%, as well as LOD and LOQs in the different matrices, providing an insight into the method’s robustness and potential for use in long-term environmental monitoring.

### Method performance

**Fig. 1:**
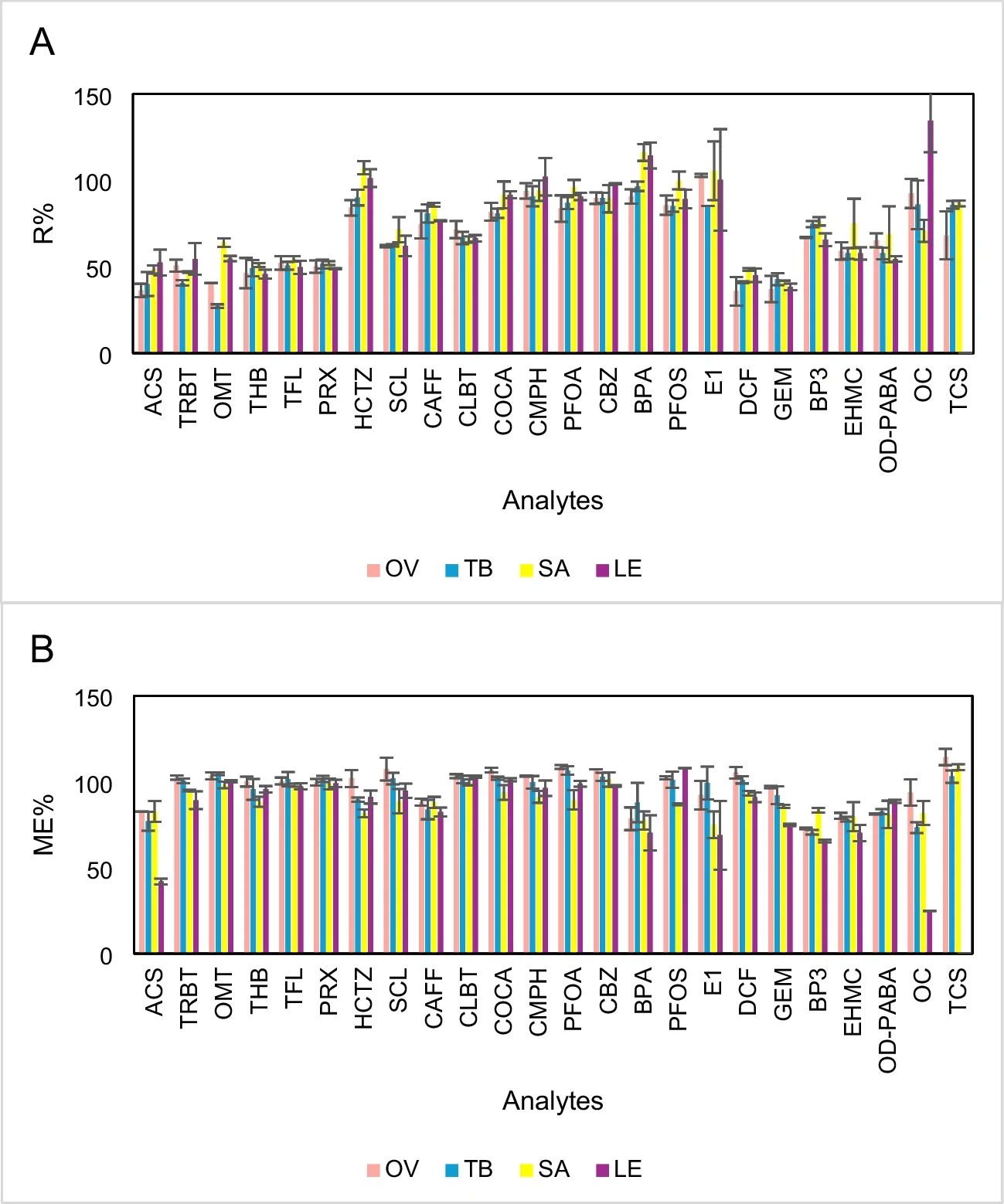
R% (**A**) and ME% (**B**) for the 23 analytes that satisfied the required criteria for *O. validus*, *T. bernacchii*, *S. antarcticus*, and *L. elliptica*

**Fig. 2:**
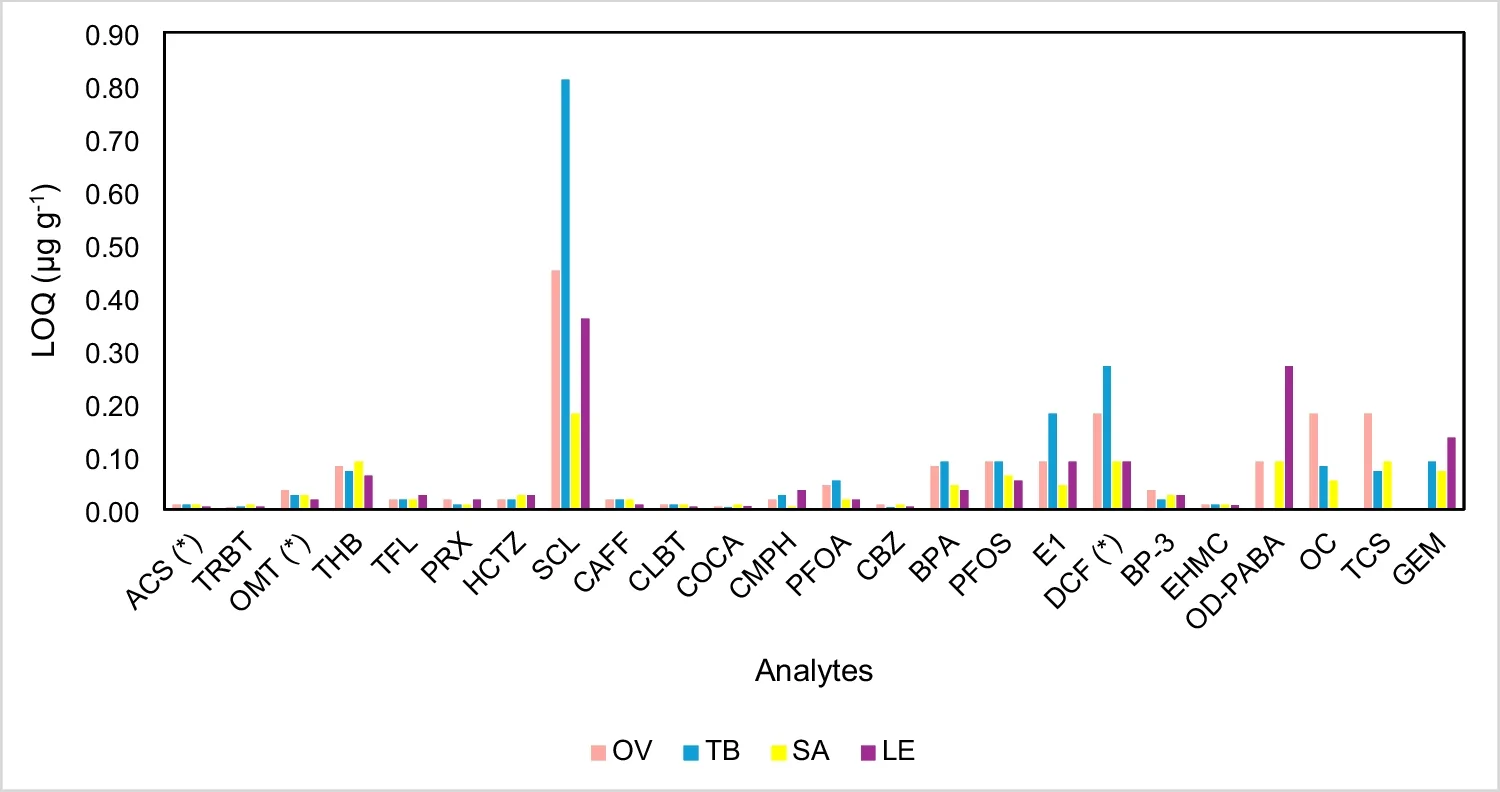
Limit of quantification for the 23 tracer pollutants, expressed as micrograms per gram for each sample of *O. validus*, *T. bernacchii*, *S. antarcticus*, and *L. elliptica*

Figure 1 shows the R% and ME% for the 23 analytes that satisfied the required performance criteria. More detailed information, including ME%, R%, and the intra- and inter-day precision (expressed as relative standard deviation, RSD%) for each of the four species is provided in the Supplementary Information (Tables S4– S7). Figure 2 is a visual representation of the obtained LOQ for each species; additional data such as the linearity range and determination coefficients can be found in Table S4, whereas LOD and LOQ details are in Tables S5– S8.

All included analytes exhibited strong linearity, with *R*^2^ values ranging from 0.997 to 0.999, in satisfactory concentration working ranges (between 0.1 and 50 µgL^−1^ for 21 compounds and between 1 and 50 µgL^−1^ for BPA and E1), confirming reliable quantification within the selected interval. LODs and LOQs, calculated as described in section “HPLC-HRMS/MS analysis,” demonstrated the method’s high sensitivity and its capability to detect low concentrations in complex biological matrices. Most recovery values of the QuEChERS procedure fell within the acceptable range of 58–116%, indicating consistent accuracy. However, deviations were observed for certain analytes: ACS, OMT, and DCF show acceptable R% only in *S. antarcticus* and *L. elliptica* but underperform in *O. validus* and *T. bernacchii* (see Tables S5 and S6). Additionally, TCS and OC were excluded from analysis in *L. elliptica* due to inadequate recovery (Table S8). THB, TFL, and PRX consistently exhibited suboptimal recoveries across all species; however, they were retained in the final method evaluation. In accordance with the SANTE 11312/2021 guidance, which allows recovery values outside the 70–120% range in exceptional cases when method performance is consistent and scientifically justified [35]. Given the rarity and limited availability of Antarctic samples, even semi-quantitative information represents an important result.

Intra-day precision, assessed through triplicate analyses, was generally satisfactory with RSD% values below 14% for most compounds, though variability was higher for *S. antarcticus*. Inter-day RSD% values, based on six replicates, were below 24% for most analytes; however, *L. elliptica* showed higher variability, with values reaching 25–33% (Table S8), suggesting matrix-dependent effects on method precision.

ME% were found to range between 66 and 114% for most compounds, indicating that interferents provoked a limited signal suppression or enhancement and/or were efficiently removed by the QuEChERS clean-up step. An exception was ACS in *L. elliptica*, which exhibited a notably low ME% of 42%, namely rather strong ion suppression.

Specificity was confirmed by acquiring two multiple reaction monitoring (MRM) transitions per analyte, and by validating the consistency of ion ratios (considered acceptable if falling between ± 30% with respect to those observed in neat standards) and retention times [36].

Although specific compounds and matrices may benefit from further optimization to enhance recovery and precision, the method overall displayed robust performance and analytical reliability for most tested analytes, showing the high versatility of the developed QuEChERS sample treatment [37–39].

### Identification of environmental pollutants through HPLC-QqQ

**Fig. 3:**
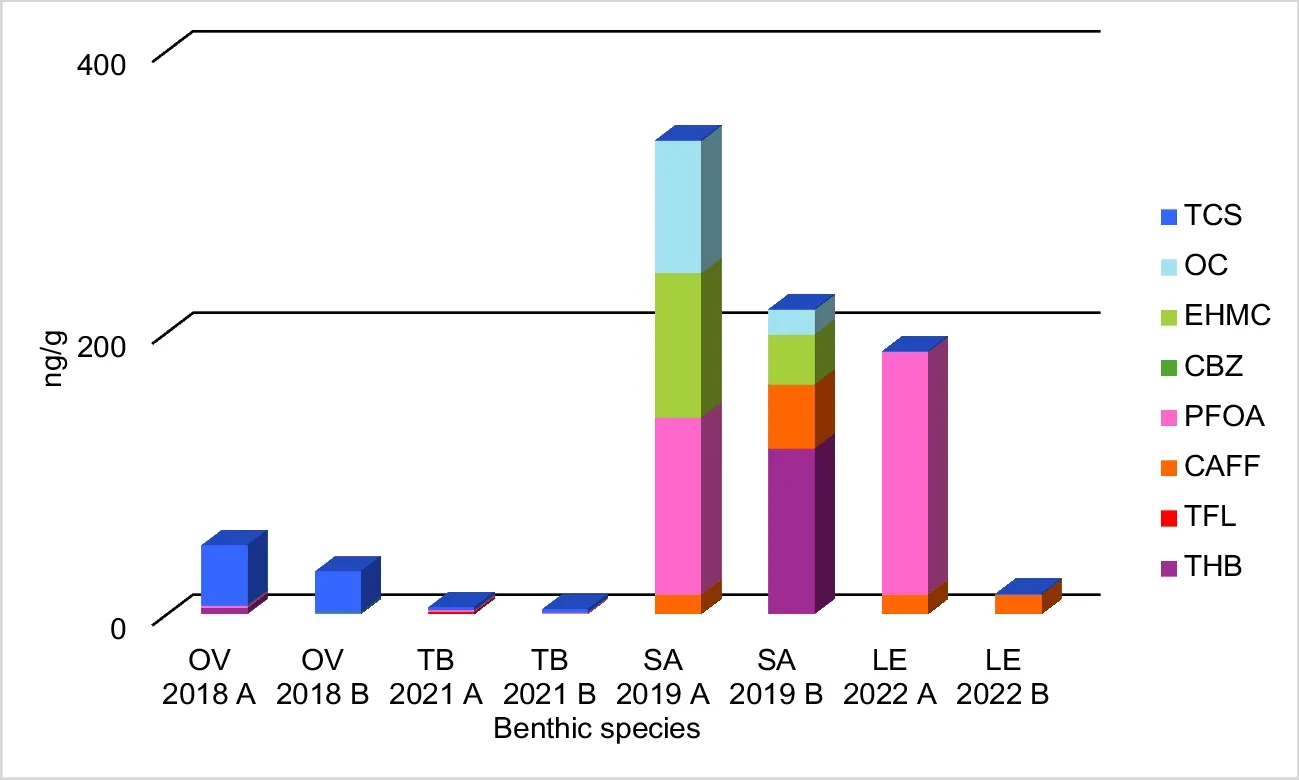
Quantitation results, expressed as nanograms per gram of dry weight in the samples *O. validus*, *T. bernacchii*, *S. antarcticus*, and *L. elliptica*. The letters A and B indicate the two procedural replicates

Over the past 20 years, chromatography coupled with tandem mass spectrometry has emerged as a leading analytical approach for the detection of ECs. Among the most widely employed techniques is LC-QqQ, favored for its broad analyte coverage and high accuracy and precision in both qualitative and quantitative analyses [40, 41]. In this study, target analysis was conducted on samples from four species, *O. validus*, *T. bernacchii*, *S. antarcticus*, and *L. elliptica*, since samples of *A. colbecki* were previously analyzed in target analysis in Gambetta Vianna (2025) et al. [25]*.* A visual representation of the results is reported in Fig. 3.

#### Tracer pollutants

Trace compounds such as CAFF, THB, and TFL are recognized as indicators of anthropogenic contamination. These compounds are usually incompletely removed during wastewater treatment processes and can persist in aquatic environments. Their presence typically originates from domestic sources, including the rinsing of coffee containers and the widespread consumption of caffeine-containing products [42]. In this study, CAFF, THB, and TFL have been found in Antarctic benthic species; results are detailed in Table S9. Among the investigated tracers, caffeine was the most frequently detected. It was above the LOD in all four *S. antarcticus* and *L. elliptica* samples but quantified only in one *S. antarcticus* specimen (below the limit of quantification in the other three samples). THB was found at trace levels in a single sample of *O. validus* and was quantified in one *S. antarcticus* specimen. TFL was detected at trace levels in *T. bernacchii*. Notably, the sample exhibiting the highest concentrations of both CAFF and THB corresponded to the *S. antarcticus* specimen.

The occurrence of caffeine in Antarctic freshwater and marine environments has been documented by Postigo et al. (2023), with mean concentrations of 37 ng L^−1^ and 57 ng L^−1^, respectively [23]. Furthermore, a recent study by MacKeown et al. (2024) reported the presence of CAFF and TFL in effluents from Mario Zucchelli Antarctic Research Station wastewater treatment plant and in passive samplers deployed in Road Bay, close to our sampling area [43].

#### Pharmaceuticals and personal care products

PPCPs represent one of the largest and most diverse categories of ECs [44]. Over the past three decades, PPCPs have been identified in nearly all environmental compartments across every continent, including remote and pristine regions such as the polar zones.

Among the wide set of PPCPs included in the targeted study, the pharmaceutical CBZ and antimicrobial agent TCS and two UV filters were detected in the investigated species.

CBZ is a tricyclic drug commonly prescribed for neurological and psychiatric conditions, including epilepsy, schizophrenia, and mood disorders [44, 45]. TCS, an antimicrobial agent, is widely incorporated into a range of PCPs, including soaps, toothpastes, deodorants, and cosmetics as well as textiles, and household items [46]. 1.1 ng g^−1^ of CBZ was quantified in *O. validus*, while TCS was found in four samples, showing a higher occurrence, consistent with previous observations in *A. colbecki* [25]. In detail, TCS was quantified in *O. validus* (29–42 ng g^−1^) and below the quantification limit in *T. bernacchii* samples. In addition to CBZ and TCS, the UV filters OC and EHMC were also detected, specifically in *S. antarcticus*. Sample A contained both compounds at quantifiable concentrations in the hundred nanograms per gram range, whereas in sample B they were detected below the quantification limit. Detailed results are provided in Table S10.

Most scientific research stations in Antarctica are situated along the coast, where domestic and industrial effluents are often discharged directly into the marine environment. Even stations equipped with WWTPs face operational challenges, particularly during the high-activity summer months when wastewater volumes increase significantly [47]. TCS has been reported in effluents and surrounding waters of multiple Antarctic stations, including Esperanza, Scott Base, McMurdo, and Mario Zucchelli Station [43, 47, 48]. The Italian Mario Zucchelli Station, located near the current sampling area, has shown consistent TCS presence in spot samples treated by solid-phase extraction (SPE), collected from its wastewater system [43]. Additionally, TCS has been detected in seawater adjacent to McMurdo and Scott Base at concentrations comparable to those observed in more densely populated coastal regions [47]. Antarctic sediment samples from Admiralty Bay, specifically Suszczewski and Herve Coves, have also shown TCS contamination, as reported by Kobusinska et al. (2020) [49].

Similarly, CBZ has been recently identified in epibenthic macroorganisms from the coastal zones of Admiralty Bay, according to Sokolowski et al. (2024) [50]. These findings suggest that benthic marine organisms in the vicinity of research stations may be exposed to PPCPs such as CBZ and TCS through direct filtration of contaminated seawater or via trophic transfer, highlighting the vulnerability of Antarctic ecosystems to even low-level, chronic pollution caused by scientific human activity. A comparable environmental pattern emerges for UV filters. In the Antarctic context, the high levels of UV radiation, resulting from the thinning ozone layers, necessitate the regular use of sunscreens and other protective formulations by personnel stationed at research bases. Consequently, there is increased environmental input of UV filter compounds in and around these facilities [51].

Previous studies have documented the occurrence of UV filters in Antarctica. The UV filter OD-PABA was identified in *A. colbecki* specimens collected from the Ross Sea in 2005, indicating the long-standing presence of such contaminants in Antarctic biota [25]. However, to the best of our knowledge, no prior studies have examined the occurrence of OC or EHMC in *S. antarcticus* or other benthic species included in the current study. The findings from Domininguez-Morueco et al. (2020) further support the environmental relevance of these compounds, revealing the OC and EHMC were among the predominant UV filters detected in suspended particulate matter from Antarctic wastewater samples, as well as in glacial meltwater. OC, in particular, exhibited the highest concentrations, likely due to its strong lipophilicity and tendency to partition into biotic and sediment matrices [52]. Complementary results from Costa et al. (2024) reported the occurrence of OC in both seawater and marine sediments in Admiralty Bay, further corroborating the widespread dispersion of these contaminants in the Antarctic marine environment [51].

#### Perfluorinated compounds

In recent years, perfluoroalkyl and polyfluoroalkyl substances (PFAS) have gained increasing scientific and regulatory concern due to their exceptional chemical stability, environmental persistence, and potential impacts on human and ecosystem health. Among these, PFOA was industrialized in the mid-twentieth century for its surfactant properties. Initially used in a wide range of applications, PFOA’s production has now been largely phased out, although PFOA remains persistent in the environment [53].

In the present study, both PFOS and PFOA were targeted for analysis. Only PFOA was quantified in six samples across all investigated species, including *T. bernacchii*, *S. antarcticus*, and *L. elliptica* specimens. Particularly elevated concentrations of PFOA were observed in *S. antarcticus* (sample A) and *L. elliptica* (detailed concentration data are provided in Table S11).

The widespread occurrence of PFOA in Antarctic biota is consistent with previous findings. Xie et al. (2020) reported PFOA as the dominant PFAS in snow collected at the Concordia Research Station (Dome C) during 2016 [54]. PFOA has been similarly detected in Southern Ocean surface waters and in Antarctic biota, including fur seal muscle and liver, as well as penguin eggs, as documented by Llorca et al. (2012) [55]. Still, the comparison with this study remains difficult due to differences in the analyzed species and reporting units (wet weight, dry weight). Discharges from WWTPs into the Antarctic Southern Sea have also been identified as a potential source of PFOA [43]. Possible local inputs in the studied area may include leaching from scientific station equipment or infrastructure, as well as emissions from WWTP effluents. The degradation of PFAS-containing materials, such as protective gear or coatings, could further contribute to the release of PFOA into the surrounding marine environment, where its persistence facilitates accumulation in both abiotic and biotic compartments.

### Tentative identification of contaminants through HPLC-HRMS and SSA

Along with the targeted analysis, a more comprehensive and exploratory suspect screening analysis was performed on the obtained extracts to investigate the contamination in benthic Antarctic species. In particular, the SSA was based on a quite large list of compounds (total 2450, including positive and negative ionization), compiled starting from different manufacturer databases and comprising several drugs, natural compounds, and PCPs. The list included analytes belonging to several categories to explore different possible sources of pollution in the Antarctic environment.

**Fig. 4:**
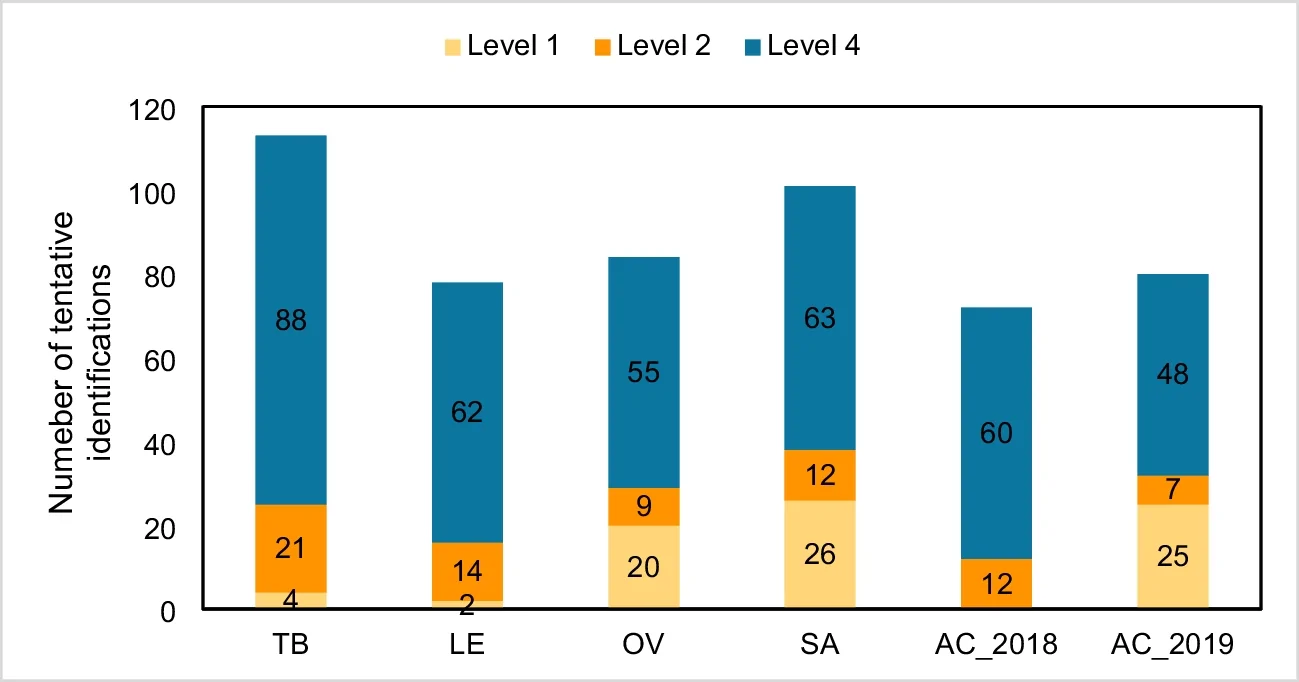
Number of tentative identifications for each sample at different confidence levels as classified by Schymanski

Figure 4 illustrates the total number of tentatively identified compounds in different benthic species, together with the associated confidence level in identification, according to Schymanski [53]. All the tentative identifications are reported in the Supplementary Material 2. In detail, 21, 14, 9, 12, 12, and 7 chemicals were assigned to level 2, in TB, LE, OV, SA, AC_2018, and AC_2019 samples, respectively, based on the match between the acquired MS and MS/MS spectra and the ones present in the used library. Among these, the compounds showing larger peak areas included the natural substances linoleic acid, linolenic acid, arachidonic acid, 9,10-epoxystearic acid, and uracil and the drug allopurinol. This observation should not be interpreted as evidence of higher concentrations [54, 55]; rather, more intense and well-defined chromatographic peaks provide higher-quality spectral information (e.g., clearer precursor ions, isotopic patterns, and product ion spectra), which supports greater confidence in the tentative identification.

A principal component analysis (PCA) was performed using the areas of the tentatively identified compounds, to investigate possible correlations among organism types and detected pollutants.

The first two principal components explained 61.2% of the total data variance, suggesting a certain degree of correlation among the detected features. The biplot shown in Figure S1 highlights similarities among several samples, with LE, OV, AC_2018, and AC_2019 displaying higher signals for a relatively limited set of features, possibly indicating the accumulation of similar chemicals in the organism. The most interesting PCA outcome, however, is the distinct positioning of the TB and SA samples in the PC1–PC2 space. TB shows very negative scores on both PC1 and PC2, whereas SA displays a strongly positive score on PC1 and a moderately negative score on PC2. Their separation from the other samples suggests that these organisms may accumulate different sets of pollutants, likely due to their distinct intrinsic biological characteristics.

## Conclusion

This study provides one of the first comprehensive assessments of ECs in Antarctic benthic species, providing a significant knowledge improvement in the polar marine ecotoxicology field. To enable the analysis, a QuEChERS-based extraction protocol, originally optimized for *A. colbecki*, was successfully applied to four additional Antarctic benthic species. The satisfactory overall method performance (accuracy, precision, and specificity) obtained for the different organisms confirmed the robustness of the approach across diverse matrices.

Targeted HPLC-QqQ analysis identified the presence of tracers, PPCPs, UV filters, and perfluorinated compounds in all species examined. *S. antarcticus* exhibited the highest contaminant burden, whereas *T. bernacchii* showed the lowest, potentially due to species-specific physiological mechanisms for contaminant elimination. Among all the analytes, PFOA emerged as the most widespread, occurring in every sample at quantifiable levels. Pharmaceuticals such as TCS, CBZ, OC, and EHMC were also detected along with tracer contaminants including CAFF, THB, and TFL. To our knowledge, this study is the first to report ECs’ presence in these Antarctic benthic species.

Subsequent suspect screening analysis via HPLC-QToF expanded the range of detectable contaminants, revealing additional potential pollutants not captured by target analysis.

Overall, the suspect screening analysis highlighted a heterogeneous contamination pattern among the investigated Antarctic benthic species. Similar accumulation profiles were observed for LE, OV, and *A*. *colbecki*, suggesting common exposure pathways or comparable ecological behaviors. In contrast, the multivariate analysis indicated the accumulation of different pollutants in TB and SA, likely related to their specific biological characteristics.

The obtained results underscore the vulnerability of Antarctic benthic fauna to contamination originating from local anthropogenic sources, including wastewater discharges from nearby research stations, tourism-related activities, and potentially long-range oceanic transport. This work demonstrates the applicability of this robust and cost-effective analytical methodology for future studies on Antarctic fauna and provides a foundation for broader assessment that may inform on the necessity for the development of regulatory frameworks for ECs in these environments.

## Supplementary Information

Below is the link to the electronic supplementary material.

ESM 1 Supplementary Material 1 (DOCX 284 KB)

ESM 2 Supplementary Material 2 (XLSX 303 KB)

## Data Availability

Data supporting the results of this study are available from the authors upon request.

## Declarations

### Statement on animal welfare

Sampling activities were conducted in accordance with internationally recognized guidelines for the protection of the environment and the welfare of Antarctic organisms.

### Source of biological material

The biological samples analyzed consist of Antarctic benthic species, collected by researchers at Jang Bogo Station in full compliance with international regulations and measures to protect local fauna.
