# Supplementary material for: Assessing emerging contaminants in Antarctic benthic marine fauna: a dual mass spectrometry investigation combining targeted and suspect screening approaches
Source: Anal Bioanal Chem. 2026 Apr 28;418(18):6033–44. doi: 10.1007/s00216-026-06512-3 (PMC13541874; doi:10.1007/s00216-026-06512-3)
Supplement: Supplementary file 1 [file 216_2026_6512_MOESM1_ESM.docx]

**SUPPLEMENTARY MATERIAL**

**Assessing Emerging Contaminants in Antarctic Benthic Marine Fauna: A Dual Mass Spectrometry Investigation Combining Targeted and Suspect Screening approaches**

Julia Gambetta Vianna^1,2^, Erica Ceccardi^1^, Barbara Benedetti^1^, Jung-Keun Oh^3^, Marina Di Carro^1^, Sarah Pizzini^2,4^, Emanuele Magi^1^

*^1^ Department of Chemistry and Industrial Chemistry, University of Genoa, via Dodecaneso 31, 1646, Genoa, Italy*

*^2^ Department of Environmental Sciences, Informatics and Statistics, Ca’ Foscari University of Venice, via Torino 155, Venezia, Mestre, Italy*

*^3^Environmental Health Research Division, National Institute of Environmental Research, Hwangyeong-ro 42, Seo-gu, Incheon 22689, Republic of Korea*

*^4^ Institute for Marine Biological Resources and Biotechnology, National Research Council (CNR-IRBIM), Largo Fiera della Pesca 2, 60125, Ancona, Italy*

**Table of content**

**Table S1:** Gradient elution conditions during the HPLC-QqQ analysis

**Table S2:** Gradient elution conditions during the HPLC-QToF analysis

**Table S3:** Details on the mass detection of all considered analytes, including the parameters of the MRM detection

**Table S4:** Determination coefficient and linearity range of the 23 analytes

**Table S5:** Matrix effect, recovery, relative standard deviation intra-day and inter-day, LOD for *O. validus*. (*) Results for this analyte should be interpreted with caution due to potential quantitative uncertainty

**Table S6:** Matrix effect, recovery, relative standard deviation intra-day and inter-day, LOD for *T. bernacchii*. (*) Results for this analyte should be interpreted with caution due to potential quantitative uncertainty

**Table S7:** Matrix effect, recovery, relative standard deviation intra-day and inter-day, LOD for *S. antarcticus*

**Table S8:** Matrix effect, recovery, relative standard deviation intra-day and inter-day, LOD for *L. elliptica*. (*) Results for this analyte should be interpreted with caution due to potential quantitative uncertainty

**Table S9:** Quantitation results of tracer pollutants, expressed as ng per g of dry weight of each sample of *O. validus*, *T. bernacchii*, *S. antarcticus*, *L. elliptica*. The letters A and B indicate the two procedural replicates. Quantification results are expressed with their relative standard deviations, calculated on the instrumental replicates

**Table S10:** Quantitation results of PPCPs, expressed as ng per g of dry weight of each sample of O. validus, T. bernacchii, S. antarcticus, L. elliptica. The letters A and B indicate the two procedural replicates. Quantification results are expressed with their relative standard deviations, calculated on the instrumental replicates

**Table S11:** Quantitation results of perfluorinated compounds, expressed as ng per g of dry weight of each sample of O. validus, T. bernacchii, S. antarcticus, L. elliptica. The letters A and B indicate the two procedural replicates. Quantification results are expressed with their relative standard deviations, calculated on the instrumental replicates

**Figure S1:** Biplot obtained with the results of the SSA

**Figure S2:** Extracted ion chromatograms of *the precursor ion and a related product ion of Allopurinol (a) and Linoleic acid (b), as representative species, in TB sample*

**Table S1:** Gradient elution conditions during the HPLC-QqQ analysis

| **Time (min)** | **A (%)** | **B (%)** | **Flow (mL min**^-1^**)** |
| --- | --- | --- | --- |
| 0.00 | 98 | 2 | 0.400 |
| 0.75 | 98 | 2 | 0.400 |
| 3.00 | 70 | 30 | 0.400 |
| 5.50 | 55 | 45 | 0.400 |
| 7.00 | 30 | 70 | 0.400 |
| 7.50 | 5 | 95 | 0.400 |
| 9.00 | 5 | 95 | 0.400 |
| 10.00 | 98 | 2 | 0.400 |

**Table S2:** Gradient elution conditions during the HPLC-QToF analysis

| **Time (min)** | **A (%)** | **B (%)** | **Flow (mL min**^-1^**)** |
| --- | --- | --- | --- |
| 0 | 98 | 2 | 0.400 |
| 1.5 | 98 | 2 | 0.400 |
| 6 | 70 | 30 | 0.400 |
| 10 | 55 | 45 | 0.400 |
| 14 | 30 | 70 | 0.400 |
| 15 | 5 | 95 | 0.400 |
| 18 | 5 | 95 | 0.400 |
| 19 | 98 | 2 | 0.400 |

**Table S3:** Details on the mass detection of all considered analytes, including the parameters of the MRM detection

| **Method with Supelco Ascentis Express® Biphenyl analytical reversed-phase column** | | | | | | | | | | |
| --- | --- | --- | --- | --- | --- | --- | --- | --- | --- | --- |
| **Analyte** | **Acronym** | **Polarity**  **(ESI)** | **Quantifier transition (m/z)** | | **CE (V)** | **frag (V)** | **Qualifier transition (m/z)** | | **CE**  **(V)** | **frag**  **(V)** |
|  |  |  | Precursor ion | Product ion |  |  | Precursor ion | Product ion |  |  |
| Acesulfame | ACS | ESI - | 162 | 82 | 12 | 94 | 162 | 78 | 36 | 64 |
| Taurine | TRN | ESI - | 124 | 79.9 | 24 | 96 | nd |  |  |  |
| Omethoate | OMT | ESI + | 214 | 155 | 12 | 68 | 214 | 125 | 20 | 68 |
| Daminozide | DMNZ | ESI + | 161 | 143 | 8 | 68 | 161 | 61 | 8 | 68 |
| Paraxanthine | PRX | ESI + | 181 | 124 | 20 | 77 | nd |  |  |  |
| Theophylline | TFL | ESI + | 181 | 124 | 20 | 77 | nd |  |  |  |
| Sucralose | SCL | ESI - | 457 | 397 | 4 | 72 | 455 | 395 | 4 | 104 |
| Caffeine | CAFF | ESI + | 195 | 138 | 20 | 100 | 195 | 110 | 20 | 100 |
| HydroChloroThiazide | HCTZ | ESI - | 296 | 269 | 16 | 170 | 296 | 205 | 20 | 170 |
| Chloramphenicol | CMPH | ESI - | 321 | 257 | 4 | 96 | 321 | 152 | 12 | 96 |
| PerFluoroOctanoic Acid | PFOA | ESI - | 413 | 369 | 5 | 40 | 413 | 169 | 20 | 40 |
| Furosemide | FRSM | ESI - | 329 | 285 | 12 | 90 | 329 | 205 | 20 | 90 |
| 2,4-dichlorophenoxyacetic acid | 2,4-D | ESI - | 219 | 160.8 | 8 | 68 | 219 | 125 | 28 | 68 |
| Carbamazepine | CBZ | ESI + | 237 | 194 | 20 | 120 | 237 | 165 | 48 | 120 |
| Metformin | MTF | ESI + | 130 | 68 | 40 | 96 | 130 | 60 | 12 | 96 |
| Atenolol | ATN | ESI + | 267 | 145 | 28 | 152 | 267 | 56 | 36 | 152 |
| Salbutamol | SLBT | ESI + | 240 | 148 | 16 | 96 | 240 | 222 | 8 | 96 |
| Chlormequat | CMQ | ESI + | 122 | 59 | 20 | 96 | 122 | 58 | 36 | 96 |
| Terbutaline | TRBT | ESI + | 226 | 152 | 16 | 96 | 226 | 107 | 32 | 96 |
| Nicotine | NCT | ESI + | 163 | 130 | 20 | 96 | 163 | 117 | 32 | 96 |
| Ketoprofen | KET | ESI +/- | 255 | 209 | 16 | 96 | 253 | 209 | 2 | 40 |
| Naproxen | NAP | ESI - | 229 | 170 | 5 | 40 | 229 | 169 | 15 | 40 |
| PerFluoroOctaneSulfonate | PFOS | ESI - | 499 | 80 | 60 | 110 | 499 | 99 | 47 | 180 |
| Metoprolol | MTPL | ESI + | 268 | 74 | 24 | 124 | 268 | 56 | 32 | 124 |
| Clenbuterol | CLBT | ESI + | 277 | 203 | 12 | 80 | 277 | 132 | 32 | 80 |
| Diclofenac | DCF | ESI - | 294 | 250 | 5 | 40 | 294 | 214 | 20 | 110 |
| Ibuprofen | IBU | ESI - | 205 | 161 | 2 | 40 | nd |  |  |  |
| BenzoPhenone-3 | BP-3 | ESI + | 229 | 151 | 16 | 116 | 229 | 105 | 16 | 116 |
| Gemfibrozil | GEM | ESI - | 249 | 121 | 4 | 110 | 249 | 106 | 50 | 40 |
| Cocaine | COCA | ESI + | 304 | 182 | 16 | 120 | 304 | 82 | 32 | 120 |
| Octyl dimethyl-p-aminobenzoate | OD-PABA | ESI + | 278 | 151 | 32 | 116 | 278 | 166 | 20 | 116 |
| EthylHexyl Methoxycinnamate | EHMC | ESI + | 291 | 161 | 16 | 60 | 261 | 179 | 4 | 60 |
| EthylHexyl Salycilate | EHS | ESI + | 251 | 139 | 4 | 76 | nd |  |  |  |
| Octocrylene | OC | ESI + | 362 | 250 | 8 | 152 | 362 | 232 | 20 | 152 |
| BisPhenol-A | BPA | ESI - | 227 | 212 | 12 | 128 | 227 | 133 | 24 | 128 |
| β-estradiol | E2 | ESI - | 271 | 145 | 40 | 180 | 271 | 183 | 40 | 110 |
| 17-α ethinyl estradiol | EE2 | ESI - | 295 | 145 | 30 | 180 | 295 | 159 | 35 | 110 |
| Estrone | E1 | ESI - | 269 | 145 | 30 | 110 | 269 | 143 | 60 | 110 |
| Triclosan | TCS | ESI - | 287 | 35 | 4 | 72 | 289 | 35 | 4 | 72 |
| Mepiquat | MPQ | ESI + | 114 | 98 | 152 | 28 | 114 | 58 | 152 | 28 |
| Theobromine | THB | ESI + | 181 | 138 | 100 | 20 | nd |  |  |  |

**Table S4:** Determination coefficient and linearity range of the 23 analytes

| **Compound** | **R^2^** |
| --- | --- |
| ACS (0.14 – 50 µgL^-1^) | 0.998 |
| TRBT (0.001 – 50 µgL^-1^) | 0.999 |
| OMT (0.003 – 50 µgL^-1^) | 0.999 |
| THB (0.02 – 50 µgL^-1^) | 0.999 |
| TFL (0.2 – 50 µgL^-1^) | 0.999 |
| PRX (0.007 – 50 µgL^-1^) | 0.999 |
| HCTZ (0.008 – 50 µgL^-1^) | 0.998 |
| SCL (0.3 – 50 µgL^-1^) | 0.996 |
| CAFF (0.09 – 50 µgL^-1^) | 0.999 |
| CLBT (0.01 – 50 µgL^-1^) | 0.999 |
| COCA (0.01 – 50 µgL^-1^) | 0.999 |
| CMPH (0.3 – 50 µgL^-1^) | 0.998 |
| PFOA (0.3 – 50 µgL^-1^) | 0.998 |
| CBZ (0.01 – 50 µgL^-1^) | 0.999 |
| BPA (0.06 – 50 µgL^-1^) | 0.992 |
| PFOS (0.003 – 50 µgL^-1^) | 0.999 |
| E1 (0.2 – 50 µgL^-1^) | 0.997 |
| DCF (0.0.06 – 50 µgL^-1^) | 0.999 |
| BP-3 (0.1 – 50 µgL^-1^) | 0.998 |
| EHMC (0.2 – 50 µgL^-1^) | 0.999 |
| OD-PABA (0.01 – 50 µgL^-1^) | 0.997 |
| OC (0.1 – 50 µgL^-1^) | 0.999 |
| TCS (0.1 – 50 µgL^-1^) | 0.999 |
| GEM (0.1 – 50 µgL^-1^) | 0.998 |

**Table S5:** Matrix effect, recovery, relative standard deviation intra-day and inter-day, LOD for O. validus. (*) Results for this analyte should be interpreted with caution due to potential quantitative uncertainty

| **Compound** | **ME (%)**  **(n=5)** | **R (%)**  **(n=5)** | **RSD (%)**  **(Intra-day, n=3)** | **RSD (%) (Inter-day, n=6)** | **LOD (µg g^-1^)** |
| --- | --- | --- | --- | --- | --- |
| ACS (*) | 82.6 ± 0.2 | 36 ± 4 | 10.7 | 10.5 | 0.003 |
| TRBT | 102 ± 1 | 51 ± 4 | 7.0 | 0.3 | 0.001 |
| OMT (*) | 103 ± 2 | 40.7 ± 0.1 | 0.3 | 23.8 | 0.01 |
| THB | 100 ± 3 | 46 ± 9 | 18.9 | 2.5 | 0.03 |
| TFL | 100 ± 2 | 52 ± 4 | 7.5 | 0.02 | 0.006 |
| PRX | 100 ± 2 | 50 ± 4 | 7.2 | 1.1 | 0.006 |
| HCTZ | 102 ± 5 | 84 ± 4 | 5.3 | 15.6 | 0.006 |
| SCL | 107 ± 7 | 62 ± 1 | 1.0 | 10.7 | 0.2 |
| CAFF | 88 ±2 | 74 ± 8 | 10.7 | 8.3 | 0.006 |
| CLBT | 103 ± 1 | 72 ± 5 | 7.0 | 5.0 | 0.003 |
| COCA | 107 ± 1 | 82 ± 5 | 6.3 | 7.7 | 0.002 |
| CMPH | 103.4 ± 0.3 | 94 ± 4 | 4.8 | 6.1 | 0.006 |
| PFOA | 109 ± 1 | 84 ± 8 | 9.1 | 3.5 | 0.02 |
| CBZ | 107.28 ± 0.05 | 90 ± 3 | 3.7 | 9.1 | 0.003 |
| BPA | 78 ± 7 | 91 ± 4 | 4.5 | 9.2 | 0.03 |
| PFOS | 102 ± 1 | 86 ± 6 | 6.5 | 13.4 | 0.03 |
| E1 | 92 ± 8 | 103 ± 1 | 0.9 | 17.9 | 0.03 |
| DCF (*) | 105 ± 3 | 36 ± 8 | 23.0 | 18.0 | 0.06 |
| BP-3 | 72.8 ± 0.4 | 67.1 ± 0.3 | 0.5 | 1.2 | 0.01 |
| EHMC | 80 ± 2 | 59 ± 5 | 8.5 | 2.4 | 0.003 |
| OD-PABA | 81.3 ± 0.4 | 65 ± 4 | 6.2 | 0.7 | 0.03 |
| OC | 94 ± 8 | 92 ± 8 | 9.2 | 12.8 | 0.06 |
| TCS | 114 ± 5 | 68 ± 14 | 20.4 | 15.2 | 0.06 |

**Table S6:** Matrix effect, recovery, relative standard deviation intra-day and inter-day, LOD LOQ for T. bernacchii. (*) Results for this analyte should be interpreted with caution due to potential quantitative uncertainty

| **Compound** | **ME (%)**  **(n=5)** | **R (%)**  **(n=5)** | **RSD (%)**  **(Intra-day, n=3)** | **RSD (%) (Inter-day, n=6)** | **LOD (µg g^-1^)** |
| --- | --- | --- | --- | --- | --- |
| ACS (*) | 77 ± 5 | 40 ± 7 | 17.5 | 7.2 | 0.003 |
| TRBT | 101 ± 1 | 41 ± 2 | 4.0 | 8.0 | 0.002 |
| OMT (*) | 105 ± 1 | 27 ± 1 | 4.0 | 33.6 | 0.009 |
| THB | 96 ± 6 | 49 ± 5 | 9.5 | 4.6 | 0.02 |
| TFL | 101 ± 4 | 51 ± 2 | 4.5 | 5.2 | 0.006 |
| PRX | 102 ± 2 | 52 ± 3 | 5.0 | 0.1 | 0.003 |
| HCTZ | 90 ± 1 | 90 ± 5 | 5.0 | 7.6 | 0.006 |
| SCL | 102 ± 3 | 62 ± 1 | 1.0 | 12.2 | 0.3 |
| CAFF | 84 ± 6 | 81 ± 5 | 6.6 | 1.6 | 0.006 |
| CLBT | 101 ± 2 | 67 ± 4 | 5.4 | 6.7 | 0.003 |
| COCA | 102 ± 1 | 81 ± 3 | 3.4 | 9.8 | 0.001 |
| CMPH | 100 ± 4 | 91 ± 6 | 6.5 | 9.1 | 0.009 |
| PFOA | 107 ± 2 | 87 ± 4 | 4.1 | 7.9 | 0.02 |
| CBZ | 103 ± 2 | 90 ± 3 | 3.1 | 10.6 | 0.001 |
| BPA | 88 ± 12 | 97 ± 3 | 2.9 | 4.3 | 0.03 |
| PFOS | 101 ± 5 | 85 ± 3 | 4.0 | 24.9 | 0.03 |
| E1 | 99 ± 10 | 85.18 ± 0.03 | 0.03 | 15.2 | 0.06 |
| DCF (*) | 101 ± 2 | 41.1 ± 0.5 | 1.2 | 12.9 | 0.09 |
| BP-3 | 71 ± 1 | 75 ± 2 | 2.4 | 6.2 | 0.006 |
| EHMC | 78 ± 1 | 58 ± 3 | 5.5 | 10.9 | 0.003 |
| OD-PABA | 83 ± 1 | 58 ± 3 | 5.8 | 8.2 | 0.0003 |
| OC | 73 ± 3 | 86 ± 14 | 16.5 | 25.1 | 0.03 |
| TCS | 103 ± 4 | 86 ± 2 | 2.5 | 5.9 | 0.02 |
| GEM | 92 ± 5 | 43 ± 3 | 8.1 | 7.3 | 0.03 |

**Table S7:** Matrix effect, recovery, relative standard deviation intra-day and inter-day, LOD for S. antarcticus

| **Compound** | **ME (%)**  **(n=5)** | **R (%)**  **(n=5)** | **RSD (%)**  **(Intra-day, n=3)** | **RSD (%) (Inter-day, n=6)** | **LOD (µg g^-1^)** |
| --- | --- | --- | --- | --- | --- |
| ACS | 83 ± 6 | 48 ± 2 | 5.5 | 9.4 | 0.003 |
| TRBT | 94.8 ± 0.4 | 47.1 ± 0.4 | 0.8 | 6.7 | 0.003 |
| OMT | 98 ± 2 | 64 ± 2 | 5.1 | 21.0 | 0.009 |
| THB | 88 ± 3 | 51 ± 1 | 2.1 | 4.1 | 0.03 |
| TFL | 98 ± 1 | 55 ± 2 | 2.7 | 3.2 | 0.006 |
| PRX | 99 ± 3 | 52 ± 1 | 2.1 | 3.1 | 0.003 |
| HCTZ | 82 ± 2 | 107 ± 4 | 4.3 | 12.4 | 0.009 |
| SCL | 89 ± 7 | 71 ± 7 | 10.0 | 0.7 | 0.06 |
| CAFF | 88 ± 3 | 86 ± 1 | 1.4 | 7.4 | 0.006 |
| CLBT | 100 ± 2 | 66 ± 2 | 2.5 | 5.5 | 0.003 |
| COCA | 94 ± 4 | 92 ± 2 | 10.5 | 15.1 | 0.003 |
| CMPH | 91 ± 3 | 94 ± 6 | 6.4 | 1.4 | 0.002 |
| PFOA | 89 ± 6 | 96 ± 4 | 4.8 | 3.5 | 0.006 |
| CBZ | 101 ± 4 | 89 ± 8 | 8.3 | 4.4 | 0.003 |
| BPA | 77 ± 5 | 116 ± 5 | 5.5 | 19.1 | 0.02 |
| PFOS | 87 ± 1 | 100 ± 5 | 5.0 | 1.4 | 0.02 |
| E1 | 75 ± 8 | 106 ± 17 | 21.6 | 20.5 | 0.02 |
| DCF | 93 ± 1 | 49 ± 1 | 1.4 | 8.5 | 0.03 |
| BP-3 | 83 ± 1 | 76 ± 2 | 4.5 | 25.5 | 0.009 |
| EHMC | 80 ± 8 | 75 ± 14 | 27.4 | 25.6 | 0.003 |
| OD-PABA | 81 ± 8 | 69 ± 16 | 40.2 | 36.4 | 0.03 |
| OC | 82 ± 7 | 71 ± 6 | 24.9 | 47.5 | 0.02 |
| TCS | 109 ± 2 | 87 ± 2 | 2.0 | 1.6 | 0.03 |
| GEM | 86 ± 1 | 42.0 ± 0.1 | 0.2 | 2.5 | 0.02 |

**Table S8:** Matrix effect, recovery, relative standard deviation intra-day and inter-day, LOD for L. elliptica. (*) Results for this analyte should be interpreted with caution due to potential quantitative uncertainty

| **Compound** | **ME (%)**  **(n=5)** | **R (%)**  **(n=5)** | **RSD (%)**  **(Intra-day, n=3)** | **RSD (%) (Inter-day, n=6)** | **LOD**  **(µg g^-1^)** |
| --- | --- | --- | --- | --- | --- |
| ACS | 52 ± 8 | 42 ± 2 | 14.3 | 10.8 | 0.002 |
| TRBT | 89 ± 5 | 55 ± 9 | 16.8 | 13.5 | 0.002 |
| OMT | 100 ± 1 | 55 ± 1 | 2.7 | 17.0 | 0.006 |
| THB | 96 ± 2 | 46 ± 3 | 5.5 | 14.1 | 0.02 |
| TFL | 97 ± 2 | 50 ± 4 | 7.6 | 18.8 | 0.009 |
| PRX | 99 ± 2 | 48.9 ± 0.3 | 0.6 | 21.5 | 0.006 |
| HCTZ | 91 ± 4 | 101 ± 5 | 5.1 | 6.0 | 0.009 |
| SCL | 95 ± 4 | 62 ± 6 | 9.6 | 9.9 | 0.1 |
| CAFF | 82 ± 3 | 76.6 ± 0.4 | 0.5 | 10.5 | 0.003 |
| CLBT | 103 ± 1 | 66 ± 2 | 2.8 | 18.7 | 0.002 |
| COCA | 101 ± 1 | 92 ± 2 | 2.3 | 2.1 | 0.002 |
| CMPH | 96 ± 5 | 102 ± 11 | 10.6 | 2.0 | 0.01 |
| PFOA | 99 ± 2 | 91 ± 2 | 2.4 | 3.2 | 0.006 |
| CBZ | 97.5 ± 0.4 | 98.1 ± 0.4 | 0.4 | 1.5 | 0.002 |
| BPA | 70 ± 10 | 114 ± 7 | 6.5 | 10.9 | 0.01 |
| PFOS | 107.96 ± 0.04 | 89 ± 5 | 6.0 | 6.6 | 0.02 |
| E1 | 69 ± 20 | 100 ± 29 | 29.3 | 9.2 | 0.03 |
| DCF | 91 ± 3 | 45 ± 4 | 8.7 | 10.2 | 0.03 |
| BP-3 | 66 ± 1 | 66 ± 4 | 5.6 | 29.9 | 0.009 |
| EHMC | 70 ± 5 | 58 ± 4 | 6.1 | 25.5 | 0.003 |
| OD-PABA | 89 ± 1 | 55 ± 2 | 2.8 | 32.8 | 0.09 |
| GEM (*) | 75 ± 1 | 38 ± 2 | 5.1 | 6.3 | 0.05 |

**Table S9:** Quantitation results of tracer pollutants, expressed as ng per g of dry weight of each sample of O. validus, T. bernacchii, S. antarcticus, L. elliptica. The letters A and B indicate the two procedural replicates. Quantification results are expressed with their relative standard deviations, calculated on the instrumental replicates

| **Analyte** | **THB**  **(ng/g)** | **TFL**  **(ng/g)** | **CAFF**  **(ng/g)** |
| --- | --- | --- | --- |
| *O. validus* A | < LOQ | ND | ND |
| *O. validus* B | ND | ND | ND |
| *T. bernacchii* A | ND | < LOQ | ND |
| *T. bernacchii* B | ND | ND | ND |
| *S. antarcticus* A | ND | ND | <LOQ |
| *S. antarcticus* B | 117 ± 13 | ND | 46 ± 2 |
| *L. elliptica* A | ND | ND | <LOQ |
| *L. elliptica* B | ND | ND | <LOQ |

**Table S10:** Quantitation results of PPCPs, expressed as ng per g of dry weight of each sample of O. validus, T. bernacchii, S. antarcticus, L. elliptica. The letters A and B indicate the two procedural replicates. Quantification results are expressed with their relative standard deviations, calculated on the instrumental replicates

| **Analyte** | **CBZ**  **(ng/g)** | **TCS**  **(ng/g)** | **EHMC**  **(ng/g)** | **OC**  **(ng/g)** |
| --- | --- | --- | --- | --- |
| *O. validus* A | ND | 42 ± 2 | ND | ND |
| *O. validus* B | 1.1 ± 0.1 | 29 ± 4 | ND | ND |
| *T. bernacchii* A | ND | < LOQ | ND | ND |
| *T. bernacchii* B | ND | < LOQ | ND | ND |
| *S. antarcticus* A | ND | ND | 102 ± 26 | 94 ± 21 |
| *S. antarcticus* B | ND | ND | < LOQ | < LOQ |
| *L. elliptica* A | ND | ND | ND | ND |
| *L. elliptica* B | ND | ND | ND | ND |

**Table S11:** Quantitation results of perfluorinated compounds, expressed as ng per g of dry weight of each sample of O. validus, T. bernacchii, S. antarcticus, L. elliptica. The letters A and B indicate the two procedural replicates. Quantification results are expressed with their relative standard deviations, calculated on the instrumental replicates

| **Analyte** | **PFOA**  **(ng/g)** |
| --- | --- |
| *O. validus* A | 1.3 ± 0.2 |
| *O. validus* B | ND |
| *T. bernacchii* A | 1.4 ± 0.2 |
| *T. bernacchii* B | 1.3 ± 0.1 |
| *S. antarcticus* A | 126 ± 4 |
| *S. antarcticus* B | ND |
| *L. elliptica* A | 172 ± 4 |
| *L. elliptica* B | 112.1 ± 0.8 |

**Figure S1:** Biplot obtained with the results of the SSA

**Figure 2:** Extracted ion chromatograms of the precursor ion and a related product ion of Allopurinol (a) and Linoleic acid (b), as representative species, in TB sample
